# Metabolic Engineering of a Serotonin Overproducing *Saccharomyces cerevisiae* Strain

**DOI:** 10.1111/1751-7915.70140

**Published:** 2025-04-05

**Authors:** Andrés Planells‐Cárcel, Elena Valera‐García, Guillermo Quintas, José Luis Martínez, Sara Muñiz‐Calvo, José Manuel Guillamón

**Affiliations:** ^1^ Departmento de Biotecnología de Alimentos Instituto de Agroquímica y Tecnología de Alimentos (IATA)—Consejo Superior de Investigaciones Científicas (CSIC) Valencia Spain; ^2^ Leitat Technological Centre Health and Biomedicine Barcelona Spain; ^3^ Department of Biotechnology and Biomedicine Technical University of Denmark (DTU) Kgs. Lyngby Denmark; ^4^ Division of Systems and Synthetic Biology Department of Life Sciences Chalmers University of Technology Gothenburg Sweden

**Keywords:** bioreactor, metabolic engineering, serotonin, yeast

## Abstract

The EU Green Deal prioritises the transformation of the chemical industry to a more environmentally sustainable model. This involves using microorganisms, such as *Saccharomyces cerevisiae* , to produce molecules more sustainably through biotechnological approaches. In this study, we demonstrate an example of serotonin production using *S. cerevisiae* as a cell factory, along with its optimisation and upscaling. To achieve this, we introduced two heterologous genes, the combination of tryptophan decarboxylase from *Clostridium sporogenes* (*Cs*TDC) and tryptamine 5‐hydroxylase from *Oryza sativa* (*Os*T5H), to complete the serotonin biosynthetic pathway using L‐tryptophan (L‐TRP) as a precursor. By modifying *ARO4* to a feedback‐resistant version (ARO4*), the flux of the shikimate pathway was significantly increased and serotonin production was achieved at levels up to 120 mg/L directly from the glucose source. After a medium optimisation, a final concentration of 80 g/L glucose and 300 mg/L of nitrogen resulted in better conditions for increasing serotonin titres. Using this medium in a 1 L bioreactor fermentation resulted in approximately 250 mg/L of serotonin. A targeted metabolomic study of the bioreactor growth medium identified potential bottlenecks in the serotonin‐overproducing strain and future targets for increasing its titre. We have constructed a strain of *S. cerevisiae* that represents the first steps towards feasible industrial production of serotonin using a sustainable and environmentally friendly approach, paving the way for the development of similar biotechnological strategies in the future.

## Graphical abstract

Yeast strain engineered for serotonin production. The medium and fermentation conditions have been optimised to achieve the highest serotonin production of any *S. cerevisiae* strain to date.

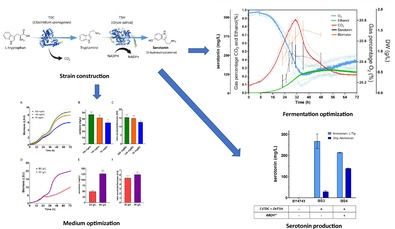

## 1 Introduction

Serotonin is a tryptophan‐derived molecule that plays an important role as a neurotransmitter in mammals, being crucial for the normal functioning of the nervous system and the immune system (Wu et al. 2019). In addition, its involvement in the microbiota‐gut‐brain axis has recently been discovered as a key factor in serotonergic signalling, as it has been shown that gut microbiota are also involved in serotonin production (Jones et al. 2020; O'Mahony et al. 2015). As a tryptophan‐derived molecule, it has an indole structure that serves as a scaffold for other bioactive molecules with interesting properties, such as melatonin, to regulate the sleep cycle (Fatemeh et al. 2022), triptans for the treatment of migraines (Cameron et al. 2015) or feruloyl serotonin and 4‐coumaryloyl serotonin, useful for use in the cosmetic industry (Kang et al. 2009; Roh et al. 2004). In addition, the serotonin molecule itself possesses antioxidant activity (Sarikaya and Gulcin 2013), which highlights its interest in industrial‐scale production of this molecule.

Serotonin is currently produced either by complete chemical synthesis (Shaw 1955) or by using a precursor molecule, 5‐hydroxytryptophan, extracted from the seeds of *Griffonia simplicifolia*, and performing chemical modifications. These processes often involve the use of organic solvents, resulting in processes that are not eco‐friendly. For this reason, biotechnological production of serotonin can be a sustainable alternative with great potential for productivity and scalability. *Saccharomyces cerevisiae* is an interesting option as a cell factory to produce this kind of indole molecules because there is a great knowledge of the biochemical pathways involved, as well as a wide variety of known molecular techniques for metabolic engineering and genomic editing (Borodina and Nielsen 2014). Furthermore, yeast's natural production of tryptophan‐derived secondary metabolites makes it particularly interesting as a cell factory (Fernández‐Cruz et al. 2017; Planells‐Cárcel et al. 2024). *S. cerevisiae* is a GRAS (Generally Recognised As Safe) organism, which facilitates its use in industry in terms of security. Several authors have used *S. cerevisiae* as a chassis for the production of tryptophan (L‐TRP) or other derived molecules (Albertin et al. 2017; Milne et al. 2020), demonstrating potential for the production of these types of molecules. In addition, using *S. cerevisiae* facilitates purification and downstream processing compared to other microbial chassis (Borodina and Nielsen 2014).

**FIGURE 1:**
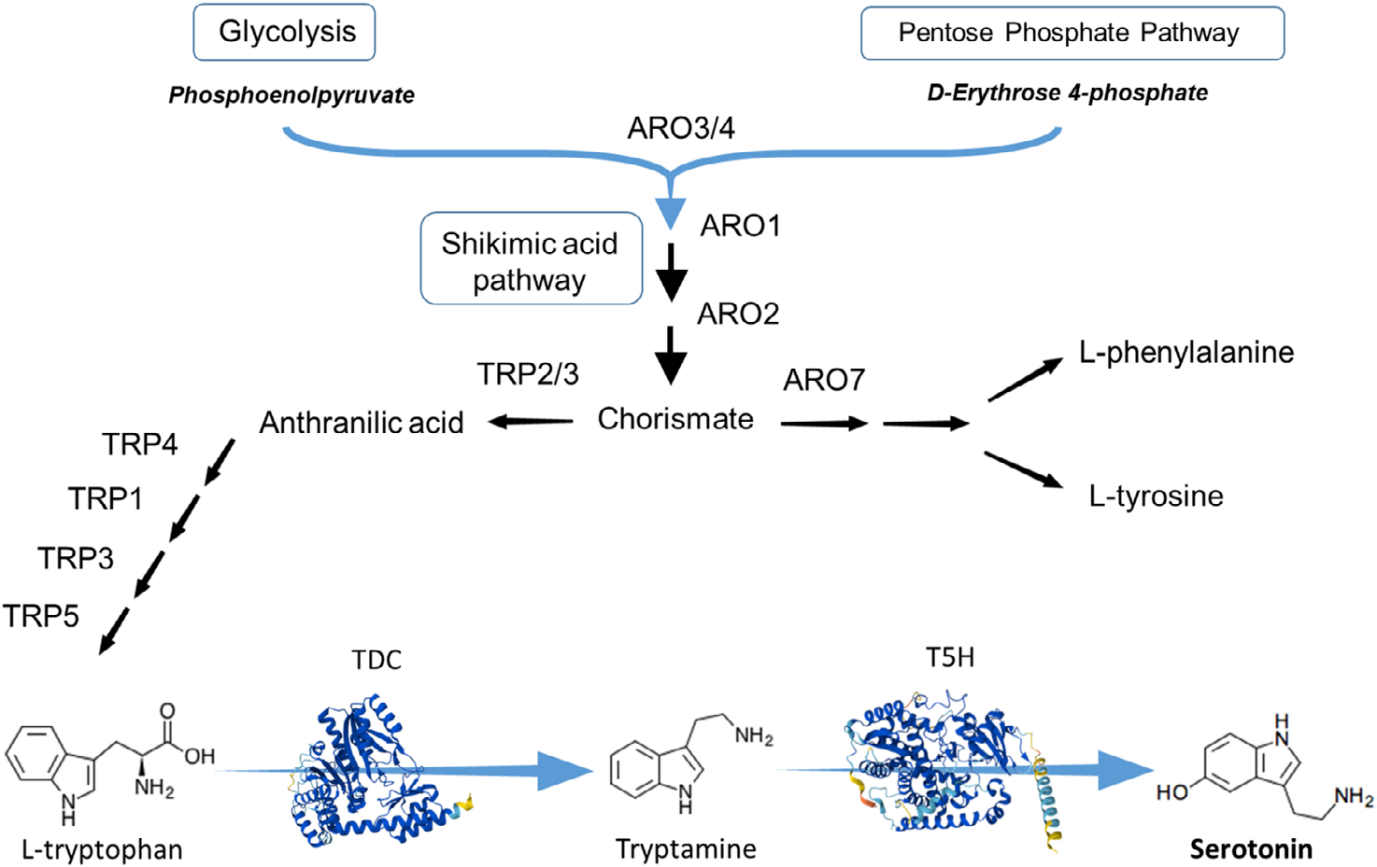
Illustrative scheme of aromatic amino acid metabolism from sugar catabolism in *S. cerevisiae* . Tryptophan is produced from the precursors phosphoenolpyruvate and D‐erythrose 4‐phosphate, from glycolysis and the pentose phosphate pathway respectively, via the shikimic acid pathway. The last part of the diagram represents the pathway we have constructed for the production of serotonin from tryptophan. The enzymes shown are tryptophan decarboxylase (TDC) from *C. sporogenes* and tryptamine 5‐hydroxylase (T5H) from *O. sativa* , represented by their AlphaFold structural models.

Serotonin biosynthesis pathway starts from L‐TRP, but depending on the organism, it can proceed in two different ways. In mammals, the most common route is first the hydroxylation of L‐TRP to 5‐hydroxytryptophan (5‐HTP), carried out by the enzyme tryptophan hydroxylase (TPH), shortly followed by the decarboxylation of 5‐HTP to serotonin, carried out by the enzyme tryptophan decarboxylase (TDC). This pathway requires cofactors that are not present in *S. cerevisiae* , as the TPH enzyme requires the tetrahydrobiopterin (BH4) along with oxygen for hydroxylation. In contrast, the most common pathway in plants is the reverse, starting with the decarboxylation of L‐TRP to tryptamine by TDC, followed by the hydroxylation of tryptamine to serotonin by tryptamine 5‐hydroxylase (T5H). This second pathway does not require the cofactor BH4 because the T5H enzyme belongs to the monooxygenase cytochrome P450 family, which needs oxygen and NADPH to add the hydroxyl group into the molecule. Cytochrome P450 enzymes are dependent on a cytochrome P450 reductase (CPR), which is involved in the electron transfer between NADPH and cytochrome P450 enzymes (Renault et al. 2014). Due to the fact that native CPR enzyme, encoded by the *NCP1* gene, already exists in *S. cerevisiae* , this pathway could be feasible in yeast (Lesuisse et al. 1997). In a previous study, we analysed the preferential production route of melatonin, the successor molecule to serotonin, in yeast (Muñiz‐Calvo et al. 2019). In this work, it was found that the preferred pathway of *S. cerevisiae* for serotonin was more similar to plants, as no L‐TRP to 5‐HTP hydroxylase enzyme activity was detected (Figure 1).

In this study, we engineered a strain capable of overproducing serotonin. Through comprehensive medium characterisation, we optimised the conditions to enhance serotonin production from glucose and scaled up the production in 1 L bioreactors. Additionally, we identified several potential targets for further metabolic engineering to improve serotonin yields.

## 2 Experimental Procedures

### 2.1 Strains and Media

The *S. cerevisiae* strains used and constructed in this study are listed in Table S1. Yeast strains were maintained in YPD medium (20 g/L glucose, 20 g/L peptone, 10 g/L yeast extract) and grown at 28°C. SD medium (1.7 g/L yeast nitrogen base without amino acids and ammonium sulphate Difco, 5 g/L ammonium sulphate, 20 g/L glucose) was used for serotonin production assays of strains with episomal plasmids. SD supplemented with L‐TRP (2 g/L) was used for the screening of transformants. SD 80 medium (80 g/L glucose, 1.7 g/L yeast nitrogen base without amino acids and ammonium sulphate) with 300 mg/L of nitrogen was used for shake flask assays. Two different nitrogen source combinations were tested in these assays. In one of these assays, the 300 mg/L of assimilable nitrogen was provided by an equimolar mixture of ammonium sulphate (0.75 g/L) and L‐TRP (2.19 g/L). In another test, the assimilable nitrogen was made up entirely of ammonium sulphate (1.41 g/L). In the case of the bioreactor tests, SD 80 was used with 300 mg/L of nitrogen in the form of ammonium sulphate only. Depending on each strain's auxotrophic needs (Table S1), media were supplemented with histidine (76 mg/L), uracil (76 mg/L) or leucine (380 mg/L).

For plasmid construction, strain *Escherichia coli* NZYa (NzyTech) was used, being cultured in LB medium (10 g/L of tryptone, 5 g/L of yeast extract and 5 g/L of NaCl) with 100 mg/L of ampicillin to maintain plasmids and grown at 37°C.

### 2.2 Plasmids and Strains Construction

All constructed plasmids and primers used in this study can be found in Tables S2 and S3, respectively. The sequences of the TDC gene from *Clostridium sporogenes* (*Cs*TDC) and T5H from *Oryza sativa* (*Os*T5H) were chemically synthesised by Twist Bioscience (California, USA) and optimised for the use of codons from *S. cerevisiae* (Table S4).

#### 2.2.1 Gene Amplification and Cloning

Synthetic genes *Cs*TDC and *Os*T5H were amplified using Phusion High‐Fidelity DNA polymerase (Thermo Scientific, Waltham, Massachusetts, USA) and the corresponding primers described in Table S3. The PCR conditions included an initial denaturation at 98°C for 30 s, followed by 30 cycles of 98°C for 10 s, 60°C for 30 s, and 72°C for 30 s per kb of target DNA, with a final extension at 72°C for 10 min. The amplified products were then digested with BamHI and XhoI restriction enzymes (Thermo Scientific, Waltham, Massachusetts, USA) following the manufacturer's recommendation. The digested products were purified using a gel extraction kit (NZYTech, Lisbon, Portugal) and ligated into the p426GPD vector (Mumberg et al. 1995) using T4 DNA ligase (Thermo Scientific, Waltham, Massachusetts, USA) at 16°C for 1 h.

#### 2.2.2 Construction of Feedback Inhibition‐Insensitive Genes

The feedback inhibition‐insensitive *ARO4* ^*K229*L^ (ARO4*) gene was obtained from a previously constructed p423GPD ARO4* vector (Bisquert et al. 2022). To construct the feedback inhibition‐insensitive *TRP2* ^*S65L S76L*^ (TRP2*), the *TRP2* ORF was first amplified from genomic DNA of the yeast strain BY4743 using the primers TRP2 BamHI F and TRP2 XhoI R (Table S3). The PCR conditions were similar to those described above. The amplified *TRP2* ORF was then cloned into the p426GPD vector (Table S2) using BamHI and XhoI restriction sites. To introduce the S65R and S76L mutations, site‐directed mutagenesis was performed using the primers TRP2 S65R F/TRP2 S65R R and TRP2 S76L F/TRP2 S76L R to introduce the S65R and S76L mutations, respectively (Table S3). Two consecutive mutagenesis processes were carried out to change each amino acid in the sequence. The mutagenesis PCR conditions included an initial denaturation at 98°C for 30 s, followed by 30 cycles of 98°C for 10 s, 61°C for 30 s, and 72°C for 30 s per kb of target DNA, with a final extension at 72°C for 10 min.

After *E. coli* transformation, colony PCR was performed using GPD_test F and CYC1_test R primers (Table S3) to screen for positive colonies using NZYTaq II 2× Green Master Mix (NZYTech, Lisbon, Portugal). Positive colonies were further verified by Sanger sequencing (Eurofins genomics, Ebersberg, Germany).

#### 2.2.3 Construction of Multiple Integration Plasmids

Multiple integration plasmids were constructed using protocols and vectors from the EasyCloneMulti system (Maury et al. 2016). Synthetic Genes *Cs*TDC and *Os*T5H were cloned in the multicopy integration plasmid pCfB2803. For this purpose, both genes and the bidirectional promoter *TEF1*p‐*PGK1*p were amplified by PCR from synthetic genes and plasmid pCfB2628 (Germann et al. 2016) (Table S2) respectively, using Phusion U Hot Start polymerase (Thermo Scientific, Waltham, Massachusetts, USA) and specific primers for USER cloning CsTDC‐GV1R, CsTDC‐GP1F, OsT5H‐GV2R, OsT5H‐GP2F, PG1R(TEF1p) and PG2R(PGK1p) (Table S3).

The feedback inhibition‐insensitive ARO4* gene was cloned into plasmid pCfB2797 HIS3, a derivative of plasmid pCfB2797 (Maury et al. 2016), changing the selection marker from *URA3* to *HIS3*. Primers PV2F (GPDp) and GV2R (ARO4) were used for this purpose. Successful cloning was confirmed by PCR with ADH1_test F and CYC1_test R primers (Table S3), followed by Sanger sequencing (Eurofins Genomics, Ebersberg, Germany).

#### 2.2.4 Yeast Transformation

The resulting integrator vectors were linearized by FastDigest NotI (Thermo Scientific, Waltham, Massachusetts, USA) and transformed into yeast using the PEG/Lithium acetate (LiAc) method (Daniel Gietz and Woods 2002). Briefly, 150 μL of yeast overnight grown cultures were inoculated into 5 mL of fresh YPD medium and incubated at 28°C until the total biomass reached 2.5 × 10^8^ cells. Cells were then pelleted, washed with LiAc (0.1 M), transferred to 1.5 mL tubes and centrifuged at maximum speed for 30 s. After discarding the supernatant, cells were resuspended with 240 μL of PEG 3350 (50% w/v), 36 μL of LiAc (1.0 M), 50 μL of single‐stranded carrier DNA (2.0 mg/mL) and 34 μL of sterile water plus 1 μg of linearized integrative vector of interest to be incorporated by the cell. After a 30 min incubation at 42°C, cells were pelleted, washed with sterile water and plated on selective media plates. To generate a control strain and remove the remaining auxotrophies from engineered strains, each strain was transformed with its respective linear product derived from the corresponding empty multiple integration plasmids (pCfB2803 for *LEU2*, pCfB2797 HIS3 for *HIS3*, and pCfB2988 for *URA3*).

### 2.3 Cultivation

#### 2.3.1 Growth Conditions for Screening of Transformants

To select the higher producer strains, purified colonies of all strains were pre‐cultured overnight on YPD medium and, after washing with distilled water, inoculated at OD_600_ 0.2 into 24‐well plates (BMG Labtech, Offenburg, Germany) with 1.5 mL of SD medium supplemented with L‐TRP. For testing allelic variations ARO4* and TRP2* in episomal plasmids, SD medium was employed. Cultures were grown for 72 h, and OD_600_ was measured. Cultures were centrifuged for 3 min at 10000× *g*, and supernatants were stored at −20°C for serotonin quantification.

#### 2.3.2 Growth Conditions for Medium Optimisation

For the microfermentations, a Biolector II was used (m2p Labs, Aachen, Germany). Precultures of the BS4 strain were prepared in 50 mL falcon tube flasks with 10 mL YPD medium and incubated at 28°C, 150 rpm, for 24 h. The cultures were grown in a 48‐well FlowerPlates (MTP‐48‐B, m2p Labs, Aachen, Germany) with a total volume of 1.5 mL. Cells from precultures were washed twice with distilled water and inoculated into the corresponding wells to obtain an initial cell concentration of 1 × 10^6^ cells/mL (OD_600_ = 0.1). For medium optimisation assays, SD medium was adjusted with varying concentrations of nitrogen and glucose, tailored to the specific conditions being tested. To evaluate the effect of different amounts of nitrogen on serotonin production, three culture media were prepared with different concentrations of ammonium sulphate: 2.83 g/L to obtain 300 mg/L of nitrogen, 1.32 g/L to obtain 140 mg/L of nitrogen and 0.52 g/L to obtain 55 mg/L of nitrogen. Each of these conditions maintained 20 g/L glucose as the sugar source. For the evaluation of the influence of sugar concentration on serotonin production, two conditions were generated: 20 g/L and 80 g/L glucose. Both conditions contained 2.83 g/L ammonium sulphate. Additionally, 20.4 g/L potassium phthalate monobasic ≥ 99.5% (Sigma‐Aldrich, Germany) was added and used as a buffer, with pH set to 5. Biological triplicates were performed in all assays. Biomass was measured as scattered light units (LSU) through the biomass filter (excitation 620 nm, emission 620 nm, gain 1). The humidity was maintained at > 85%, the temperature was set at 28°C, and the stirring speed was set at 1000 rpm with a shaking diameter of 3 mm, with data being taken during 72 h. The specific growth rate values (μ_max_) were calculated based on the LSU data for all the conditions.

To determine serotonin production after medium optimisation, BY4743, BS3 and BS4 strains were inoculated in 5 mL of YPD medium and grown overnight at 28°C with orbital shaking at 300 rpm. A cell count of 2 × 10^6^ cells/mL (OD_600_ = 0.2) was used to inoculate in SD 80 medium with 300 mg/L of nitrogen in two different nitrogen conditions: one with all nitrogen sources from ammonium sulphate, and one where there was a 1:1 ratio between ammonium sulphate and L‐TRP. The strains were inoculated into Erlenmeyer flasks with a ratio of medium volume/total volume of 1/5. This culture was incubated under constant agitation (150 rpm) at 28°C for 72 h. Final OD_600_ was measured, 1.5 mL of cultures were centrifuged for 3 min at 10000× *g* and supernatants were stored at −20°C for HPLC‐FLD analysis.

#### 2.3.3 Bioreactor Cultivations

Batch fermentations with BS4 strain were performed on 1 L Biostat Qplus bioreactors (Sartorius Stedim Biotech, Germany) equipped with measurement probes for pH, dissolved oxygen (DO) and temperature. The culture medium employed was SD 80 with 300 mg/L of nitrogen in the form of ammonium sulphate. Antifoam 204 (Sigma Aldrich, Germany) at 0.3 mL/L was added to the medium. Precultures were overnight grown in a 500 mL shake flask with 50 mL YPD incubated at 28°C and 150 rpm. Cells were washed twice with distilled water and inoculated into the bioreactor at a cell density of 2 × 10^6^ cells/mL (OD_600_ = 0.2) in a final volume of 1 L. The temperature of the bioreactor was kept constant at 28°C, the agitation was set at 300 rpm and the pH was controlled to 5 by automatic addition of base (2 M NaOH). A DO probe (Model OxyFerm‐FDA 160, Hamilton) was used to measure the DO concentration, and a pH probe (Model EasyFerm Plus K8 160, Hamilton) was used for pH. The volumetric flow rate of air was set at 0.75 vvm (1 volume of air per volume of medium per minute). During fermentation, off‐gas CO_2_, O_2_ and ethanol were monitored continuously (Prima BT MS, Thermo Scientific, Waltham, Massachusetts, USA), and data acquisition was achieved using the Lucullus software (Securecell AG, Urdorf, Switzerland). Fermentations were performed in biological triplicates.

### 2.4 Analysis of Metabolite Production

#### 2.4.1 Serotonin Analysis by HPLC‐FLD

Serotonin was detected by high‐performance liquid chromatography (HPLC) on a Waters ACQ Arc Sys Core chromatograph (Waters, Milford, MA, USA), using an Accucore C18 (Thermo Scientific, Waltham, Massachusetts, USA) reversed‐phase column, with dimensions of 4.6 × 150 mm and a 2.6 μm particle size. Samples were diluted to 50% with HPLC‐quality absolute methanol and filtered with 0.22 μm nylon filters prior to injection. The mobile phases used were A (acetonitrile) and B (0.01% formic acid in water) with a constant flow rate of 0.8 mL/min; the injection volume was 10 μL and the gradient programme was set as follows: initial flow rate of 5:95% (A:B) 0–7 min, 90:10% (A:B) 7–11 min, 5:95% (A:B) 11–17 min. Analytic detection took place on a Waters 2475 fluorescence detector (FLD) where the chromatogram corresponding to λ excitation = 295 nm and λ emission = 330 nm was extracted. The retention time for serotonin in this method was 5.5 min.

#### 2.4.2 Metabolic Indole Analysis

Samples were thawed at room temperature, vortexed for 15 s, and centrifuged at 10000 x *g* for 15 min at 4°C. Following this, 50 μL of each sample was transferred to a 1.5 mL tube and mixed with 50 μL of an internal standard solution. The mixture was vortexed for another 15 s and then transferred to a 96‐well plate for analysis. The analysis of several indole compounds (L‐TRP, 5‐HTP, tryptamine, serotonin, N‐acetylserotonin, 5‐metoxytriptamine, melatonin, N‐formylkynurenine, L‐kynurenine, kynurenic acid, xanthurenic acid, L‐3‐hydroxykynurenine, 3‐hydroxyanthranilic acid, anthranilic acid, quinolinic acid, L‐phenylalanine, L‐tyrosine) was performed using UPLC‐MSMS on an Acquity‐Xevo TQS system (Waters, Milford, USA) with electrospray ionisation. This method was based on a previously validated method (Lario et al. 2017) using the MRM conditions described below: L‐3‐hydroxykynurenine 225.1 > 110; 5‐HTP 221.1 > 162.2; 5‐HTP (D4) 225 > 208; serotonin 177 > 115; serotonin (D4) 181 > 164; L‐kynurenine 209 > 94; L‐kynurenine (D4) 213 > 98; N‐formylkynurenine 237.1 > 136; L‐phenylalanine 166.1 > 91; 3‐hydroxyanthranilic acid 153.9 > 80; L‐TRP (D5) 210 > 193; L‐TRP 205 > 118; xanthurenic acid (D4) 210 > 164; xanthurenic Acid 206.1 > 132; kynurenic acid 190 > 89; kynurenic acid (D5) 195 > 149; tryptamine (D4) 165 > 148; tryptamine 161 > 122; anthranilic acid 137.89 > 120; melatonin (D4) 237 > 178; melatonin 233.17 > 159; L‐tyrosine 182.1 > 91.0.

Analysis of the samples was performed using an Acquity HSS T3 C18 column (100 × 2.1 mm, 1.8 μm). The mobile phases were water (0.1% v/v formic acid) (A) and acetonitrile (0.1% v/v formic acid) (B). The elution gradient was as described below: phase B was maintained at 2% from 0 to 0.5 min, then linearly increased to 45% over 5 min. The B‐phase was then increased to 90% in 0.2 min, immediately followed by a rapid return to initial conditions between 5.7 and 6 min, which was maintained for 1.5 min for re‐equilibration of the column. Injection volume was 3 μL, flow rate of 550 μL/min and column temperature 55°C. Autosampler temperature was set at 6°C during sample analysis. Electrospray ionisation was conducted using the following conditions: 2.9 kV capillary, 25 V cone, source temperature of 120°C, desolvation temperature of 395°C, N_2_ flow rate of the cone was 150 L/h and desolvation gas was 800 L/h.

### 2.5 Statistics

Student's *t*‐test was performed to determine the level of significance on pairwise comparisons between modified strains relative to the control strain. Statistical significance level was set at *p*‐value *p* ≤ 0.05. One Way ANOVA analysis was used, corrected for multiple comparisons using Tukey test (*p* ≤ 0.05 confidence) for comparisons between control, BS3 and BS4 strain with respect to tryptophan‐derived metabolites. Statistical significance was shown as asterisks (* = *p* ≤ 0.05; ** = *p* ≤ 0.01; *** = *p* ≤ 0.001). Analysis and figures were made by GraphPad Prism 7.0 (GraphPad Software, San Diego, CA, USA).

Regression analysis was performed to calculate the μ_max_ values of microfermentations and bioreactor fermentations using Microsoft Excel 2016, using a coefficient of determination (*R* ^2^) of above 95% as statistically significant.

## 3 Results and Discussion

### 3.1 Establishing a Platform Strain for Serotonin Biosynthesis: Selection of Heterologous Genes

In a recent study from our group (Muñiz‐Calvo et al. 2019), we proposed that serotonin in *S. cerevisiae* is predominantly formed via L‐TRP decarboxylation followed by tryptamine hydroxylation, as in plants. For this reason, we decided to use a TDC and a T5H in this order to simulate natural conditions in yeast and promote production (Figure 1), and to avoid the addition of cofactors and recycling routes (Germann et al. 2016).

After deciding on the pathway to insert in the cell, we conducted a bibliographic search to select a TDC and T5H enzyme with the best catalytic activity and good expression in yeast. For the TDC enzyme, Williams et al. (2014) discovered a novel tryptophan‐specific decarboxylase activity in *Clostridium sporogenes* , which decarboxylates L‐TRP to form the β‐arylamine neurotransmitter tryptamine.

In the case of T5H, this enzyme does not require cofactors, as was the case with *Homo sapiens* tryptophan hydroxylase (TPH) used by Germann et al. (2016), which required the introduction of the BH4 biosynthesis and regeneration pathways to avoid the need to add this cofactor to the growth medium. It had previously been discovered that the T5H enzyme from rice ( *Oryza sativa* ) was able to hydroxylate tryptamine to serotonin. T5H was previously cloned and overexpressed in *E. coli* , which was able to produce serotonin in combination with a TDC, but at a lower yield of approximately 24 mg/L (Park et al. 2011), perhaps because the family of cytochrome P450 monooxygenases is difficult to clone in *E. coli* and does not always function directly due to its transmembrane domain (Larson et al. 1991). This problem does not occur in *S. cerevisiae* , which can correctly express the full complement of cytochromes P450 and, therefore, could have a good expression of the *Os*T5H protein.

For optimal expression of the selected enzymes, chemical synthesis of both genes was chosen to change the nucleotide sequence and to optimise codon usage for *S. cerevisiae* . Codon‐optimised versions of *Cs*TDC and *Os*T5H were used for integration into the yeast genome under the expression of the strong *TEF1*p and *PGK1*p promoters, respectively. In addition, we chose gene integration rather than episomal plasmid overexpression for the sake of stability and maintenance of the integrated information through cell divisions (Maury et al. 2016). Therefore, the genes of interest were integrated in TY retrotransposon locations, a family of transposable elements distributed throughout the genome (Cameron et al. 1979), which allowed us to generate multiple insertions of several copies of our cassette of interest. Previous works have reported the use of this type of plasmid for the production of hydroxytyrosol or psilocybin, demonstrating its benefits for gene overexpression and production of compounds of interest (Bisquert et al. 2022; Milne et al. 2020). Accordingly, we introduced *Cs*TDC and *Os*T5H via integrative vector pCfB2803 targeting yeast retrotransposon Ty4 sites.

The derived strains were assessed for serotonin production. Twenty‐four colonies were screened after cultivation in SD medium enriched with L‐TRP (Figure S1). The colony exhibiting the highest serotonin production (230 mg/L) was selected and designated as strain BS1.

### 3.2 Engineering the Shikimate Pathway for Enhanced Synthesis of Serotonin From Glucose

The shikimate pathway is a major node in the production of amino acids and other related metabolites which serve as a building block molecules for a wide range of secondary metabolites derived from aromatic amino acids (Averesch and Krömer 2018). A key point in constructing a serotonin‐overproducing strain for industrial use is to achieve its production from a simple and economical source such as glucose. Additionally, precursor supply is crucial in cell factories and can significantly impact the production of the desired compound. Therefore, to facilitate serotonin biosynthesis, the strain was modified to target the shikimate pathway, increasing the metabolic flux to L‐TRP from glucose. A deregulated variant of the *ARO4* protein (*ARO4* ^K229L^ − ARO4*; Fukuda et al. 1991), which triggers an increase in the flow of the shikimic acid pathway, was explored. This mutation has been reported to be successful in increasing the L‐TRP pool in high quantities, allowing this increase in amino acids to be used by heterologous pathways (Bisquert et al. 2022; Milne et al. 2020; Zhang et al. 2020). We also found that in the literature there was a mutated allele of *TRP2* (*TRP2* ^S65R,S76L^) that creates feedback‐resistant enzyme forms that could help to increase the flux to L‐TRP (Graf et al. 1993).

A first trial to prove if these allelic variants could increase serotonin production directly from glucose involved overexpressing these allelic variants in episomal plasmids, using p426GPD TRP2* and p423GPD ARO4*. Both plasmids were transformed individually and in combination inside the BS1 strain, generating the strains BS1‐T, BS1‐A, BS1‐TA, and their serotonin production was analysed in comparison with the BS1 strain (Figure S2). A significant increase in serotonin production was observed in the BS1‐A strain in comparison to the BS1 and BS1‐T strains, which also showed an increase but with less significant results. The strain BS1‐TA, with a combination of both mutated allelic variants, shows no significant increase compared to the BS1‐A strain; therefore, we decided to integrate in multiple copies only the ARO4* variant. Hence, we integrated the ARO4* under the control of the GPD promoter in multi‐copy via the expression vector pCfB27973 HIS3 targeting yeast retrotransposon Ty2 sites. To obtain a colony with a high serotonin titre, the same procedure as for the selection of the BS1 strain was used. Twenty‐four colonies were randomly screened for serotonin production by HPLC‐FLD measurements after cultivation for 72 h in SD medium enriched with L‐TRP. Among them, a clone that produced around 240 mg/L of serotonin was selected and named BS2 (Figure S3).

Despite all the modifications made to BS2, the strain remained auxotrophic for uracil. To eliminate the need for uracil supplementation in the medium, the BS2 strain was transformed with the empty plasmid pCfB2899, creating the BS4 strain, which complements the uracil auxotrophy and allows growth in minimal medium. For the purpose of comparing production between strains with and without ARO4*, the same procedure was followed on the strain BS1, transforming it with pCfB2797 HIS3 and pCfB2899 to complement auxotrophies *HIS3* and *URA3* respectively, creating the BS3 strain. Studies have shown that differences between genetic complementation and nutritional supplementation of auxotrophic yeast strains, including *URA3* auxotrophic strains, can exhibit different growth rates and metabolic profiles, which can affect the yield and stability of heterologous protein production (Pronk 2002). Interestingly, neither growth nor serotonin production was affected by the integration of *URA3* into BS2 and *HIS3* and *URA3* into BS1, as no significant differences in serotonin production were detected (Figure S4).

### 3.3 Optimisation of the Medium for Serotonin Production

**FIGURE 2:**
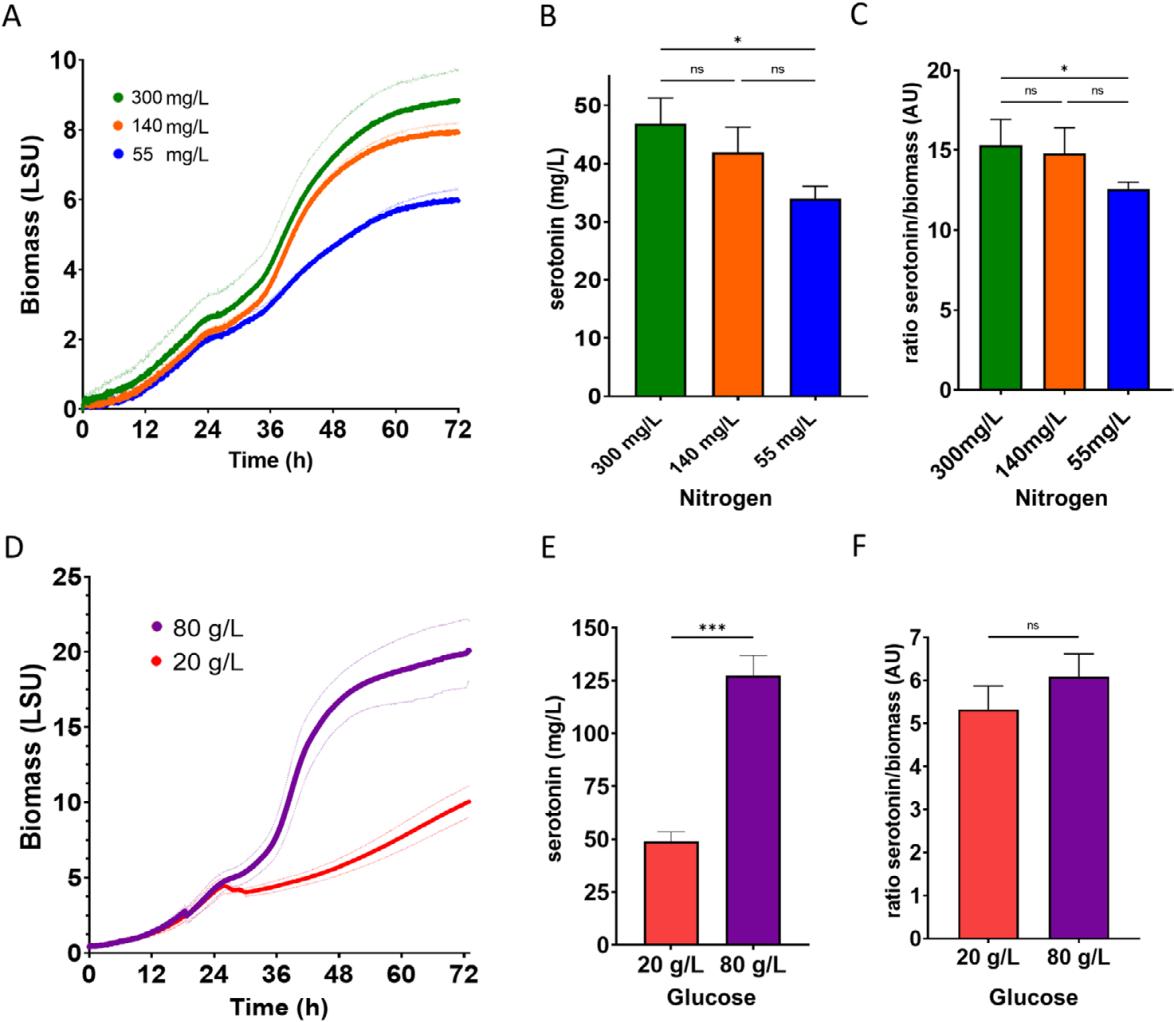
Effect of nitrogen and glucose concentration on biomass, measured as Light scattering (LSU) and serotonin production (mg/L). (A) Growth curve of strain BS4 at high (green), intermediate (orange) and low (blue) nitrogen concentration in the media. (B) Serotonin production in each of the nitrogen conditions evaluated. (C) Ratio between serotonin and biomass for each nitrogen condition evaluated. (D) Growth curve of strain BS4 at low (red) and high (purple) glucose concentration in the medium. (E) Serotonin production in each of the glucose conditions evaluated. (F) Ratio of serotonin to biomass for each glucose condition tested. Assays were performed by biological triplicate. AU, arbitrary units. Asterisks indicate the significance of the difference between each condition according to a *t*‐test statistic, that is, with less than a *p*‐value of 0.05 (*) or less than a *p*‐value of 0.01 (***), ns = not significant.

The composition of the fermentation conditions plays a key role in the growth of the yeast and can directly influence the biosynthesis of the final products. In order to explore suitable fermentation conditions for our engineered strain BS4, various concentrations of glucose and nitrogen were evaluated (Figure 2). The rationale for choosing these initial concentrations of glucose and nitrogen came from our results in previous studies on the overproduction of hydroxytyrosol, a very interesting bioactive molecule also derived from the metabolism of aromatic amino acids (Muñiz‐Calvo et al. 2020; Bisquert et al. 2022).

First, we studied the effect of nitrogen concentration in relation to serotonin production (Figure 2A–C). For this purpose, we conducted several cultures with different amounts of total nitrogen by using a high throughput microbioreactor that enables real‐time evaluation of biomass in 1.5 mL of volume. The nitrogen source used was ammonium, since it is a cheap source frequently used in the industry and easy to assimilate by yeast. Specifically, we evaluated high (300 mg/L), intermediate (140 mg/L) and low (55 mg/L) nitrogen concentrations in SD medium with 20 g/L of glucose.

The growth profiles based on light scattering (LSU) of strain BS4 in each condition were monitored (Figure 2A). We did not observe any significant change in growth before 24 h for high, intermediate and low nitrogen, exhibiting all conditions a μ_max_ around 0.124 h^−1^ (μ_max_ = 0.1151 ± 0.027 h^−1^; μ_max_ = 0.1332 ± 0.026 h^−1^; μ_max_ = 0.1405 ± 0.026 h^−1^ for high, intermediate and low nitrogen respectively). However, after 24 h BS4 exhibited a slowdown in growth. The strains growing in high nitrogen and intermediate conditions were the ones that had higher μ_max_ (0.0652 ± 0.013 h^−1^ and 0.0746 ± 0.016 h^−1^ respectively), reaching higher biomass. In contrast, the strains grown in low nitrogen condition showed a lower growth (μ_max_ = 0.0471 ± 0.009 h^−1^) and reached a 25% less biomass than in high nitrogen condition. As shown in Figure 2B, serotonin production was affected by nitrogen concentration. The highest serotonin titre (47 mg/L) was achieved under conditions of high nitrogen content, indicating a positive correlation between nitrogen availability and serotonin production. Intermediate and low nitrogen levels resulted in correspondingly lower serotonin titres (42 mg/L and 34 mg/L, respectively). No significant differences were observed between high and intermediate nitrogen conditions, even when serotonin titre was normalised to biomass (Figure 2C). However, a clear significant decrease in both serotonin titre and serotonin/biomass ratio was observed for the low nitrogen condition. Based on these results, we decided to set 300 mg/L as the optimal nitrogen condition in the subsequent experiments.

In our previous work, we showed that glucose concentration influenced the production of amino acid‐derived secondary metabolites (Bisquert et al. 2022). Hence, we evaluated the effect of glucose in the medium on serotonin production (Figure 2D–F). We tested 20 g/L and 80 g/L glucose in SD medium with 300 mg/L of nitrogen and monitored the growth of the BS4 strain over 72 h (Figure 2D). Initially, both strains showed a similar growth profile (μ_max_ of 0.09456 ± 0.0216 h^−1^ and 0.10041 ± 0.0252 h^−1^), but after 24 h, the BS4 strain growing in higher sugar concentration showed a faster rate (μ_max_ of 0.0993 ± 0.0185 h^−1^) compared to the strain in low sugar concentration (μ_max_ of 0.0192 ± 0.0214 h^−1^). Both reached their maximum biomass at around the 72 h fermentation time. As can be observed in Figure 2D, the BS4 strain growing with 80 g/L glucose presented higher biomass, reaching more than twice as much as the strain in the 20 g/L glucose condition. The growth of the BS4 strain in 20 g/L of glucose produced 50 mg/L of serotonin, whereas serotonin increased significantly to 120 mg/L under conditions of 80 g/L glucose, accompanied by enhanced cell growth (Figure 2E). Thus, the increase in serotonin was proportional to the increase in biomass with a slightly higher ratio but not significant when we compared the serotonin/biomass ratio between strains (Figure 2F). Nevertheless, this indicated that higher sugar levels enhance biomass and serotonin production. Consequently, the 80 g/L glucose condition was chosen as a parameter for culture medium optimisation in the next experiment.

**FIGURE 3:**
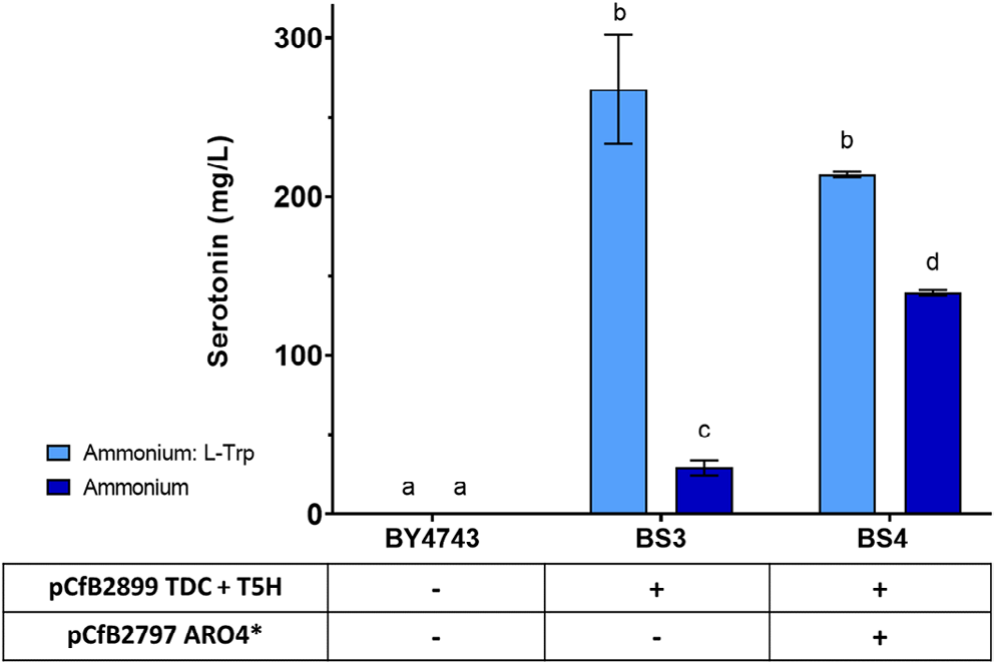
Effect of expressing *Cs*TDC, *Os*T5H and ARO4* in BY4743 on serotonin production. Table below the graph list heterologous genes present in the different strains. BS3 overexpresses the *Cs*TDC and *Os*T5H genes, BS4 overexpresses *Cs*TDC, *Os*T5H and ARO4*, wild type BY4743 was used as a control. Strains were cultivated for 72 h in SD 80 medium with 300 mg/L of nitrogen in the form of ammonium sulphate and L‐TRP mixture (light blue) or ammonium sulphate exclusively (dark blue). Standard deviations from the values of three biological replicates are represented by error bars. The serotonin titres produced by the different strains and the two media were compared using one‐way ANOVA and Tukey's test for a *p*‐value < 0.05. Shared letters indicate no significant difference in serotonin titres.

To summarise, we evaluated the BS3 and BS4 strains under the optimal conditions we selected, 80 g/L glucose and 300 mg/L of nitrogen. For this purpose, the nitrogen source was either coming exclusively from ammonium form, as had been evaluated previously on medium optimisation assays, or from a mixture of ammonium and L‐TRP in equal amounts of nitrogen concentration, as was used on colony selection. Figure 3 summarises the different serotonin titres for the wildtype BY4743, BS3 (harbouring *Cs*TDC and *Os*T5H genes for serotonin production) and the BS4 strain (BS3 strain background plus ARO4* gene) after 72 h of growth.

As illustrated in Figure 3, strain BS3 exhibited the highest serotonin production, approximately 270 mg/L, when a mixture of ammonium and L‐TRP was used as the nitrogen source. This production was about nine times higher than the production achieved using only ammonium (~29 mg/L). External supplementation with L‐TRP can effectively increase the production of tryptophan‐derived compounds by providing readily available precursors, thereby bypassing some of the regulatory and metabolic limitations of the endogenous biosynthetic pathway, as has been demonstrated for other tryptophan‐derived molecules (Li et al. 2024; Reed et al. 2024). Unexpectedly, the titre of serotonin was significantly reduced to 214 mg/L in strain BS4 in the mixed nitrogen source medium with L‐TRP. This decrease in serotonin production when L‐TRP is present in the strain overexpressing multiple copies of ARO4* could be explained by some kind of feedback retroinhibition by L‐TRP or induction of other enzymes such as *ARO7* and *TRP2* (Schmidheini et al. 1990; Zalkin et al. 1984), which redirect metabolic flux towards the phenylalanine and tyrosine pathways, reducing the amount of L‐TRP and consequently slightly reducing the capacity to produce serotonin. Nevertheless, when only ammonium was used, the production of serotonin by strain BS4 (139 mg/L) represented a 5‐fold increase compared to BS3, indicating a critical role of ARO4* overexpression in enhancing serotonin production in the absence of L‐TRP by increasing the chorismate pool from glucose.

From an industrial perspective, using ammonium as a nitrogen source is generally more feasible than using a mixture of ammonium and L‐TRP. Ammonium salts are typically cheaper and more readily available than L‐TRP, making them more cost‐effective for large‐scale production. Therefore, the BS4 strain is more interesting from an industrial perspective to produce recombinant serotonin.

### 3.4 Optimisation of Serotonin Titre Produced by Batch Fermentation in 1 L Bioreactors

**FIGURE 4:**
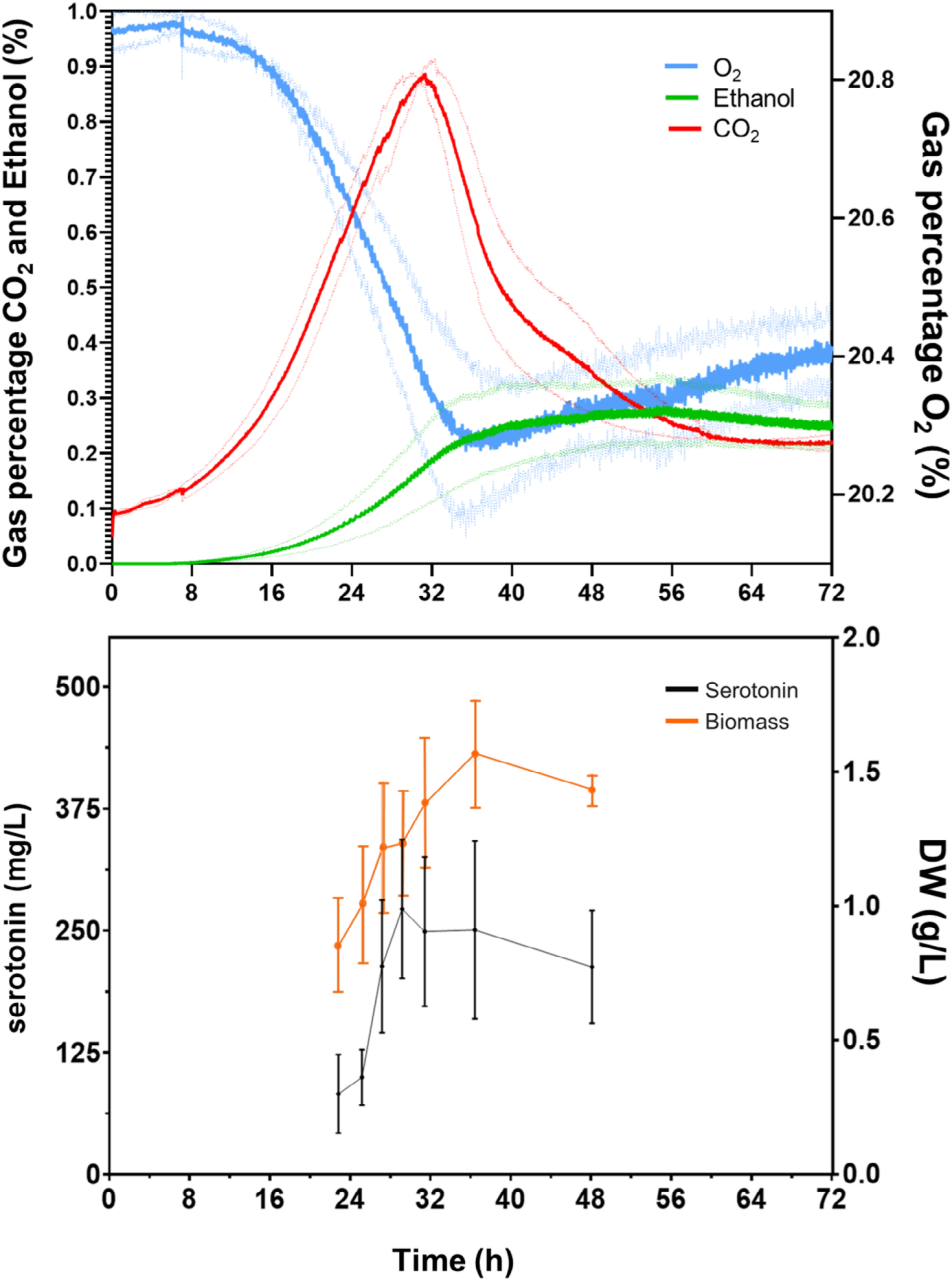
Fermentation in 1 L bioreactor of BS4 strain. Medium of culture was SD 80 with 300 mg/L of nitrogen as ammonium sulphate. The gas percentages (%) of O_2_ (blue), CO_2_ (red) and ethanol (green) were taken in real time from the gas outlet. Dashes lines represent error bars of the three replicates. Serotonin production (black) and biomass (orange), measured as dry weight (DW), were measured in the samples at 22, 25, 27, 27, 29, 31, 26 and 48 h. Data is represented as mean and standard deviation based on three replicates.

To demonstrate the industrial application of the BS4 strain as a serotonin cell factory, a batch fermentation of 1 L volume was performed. The fermentation was conducted in 1‐L bioreactors in triplicate using SD medium with 80 g/L glucose and 300 mg/L of nitrogen from an ammonium source. By using this system, we were able to control key parameters such as pH, agitation or oxygen supply and to guarantee a better control of the whole process compared to microtiter plates or shake flask fermentation. Throughout the 72‐h fermentation, off‐gas percentages of O_2_, CO_2_ and ethanol produced were measured in real time through the connection of this output to a mass spectrometer. Sampling was carried out at 22, 25, 27, 29, 31, 26 and 48 h to calculate the dry weight biomass (DW) and the serotonin produced (Figure 4).

As illustrated in Figure 4, at 32 h, CO_2_ peaked, followed by a drop that stabilised after 48 h. This point indicates the end of the exponential phase, where the biomass is stabilised and the cells switch to the stationary phase. The μ_max_ reached was about 0.1156 ± 0.0235 h^−1^. If we examine the biomass profile, we can observe that it reached its maximum at 36 h, as opposed to the 72 h required in the optimisation assays carried out in microfermenters. This higher biomass yield could be explained by the larger volume and better homogenisation of the medium or better access to the oxygen in the bioreactor. Nevertheless, the maximum titre of extracellular serotonin was 250 mg/L, reached at 36 h, which exceeded the 140 mg/L previously obtained in the shake flash assay, being the highest concentration of serotonin obtained using only ammonium as the nitrogen source. The fermentation parameters resulting from this assay correspond to a yield of 3.125 mg/g glucose and a serotonin productivity of 6.94 mg/L/h, being an interesting beginning to establish a yeast strain as an industrial platform for serotonin production.

### 3.5 Identification of Metabolic Bottlenecks Using Targeted Metabolomics Techniques

During metabolic engineering of yeast for heterologous production of metabolites, metabolic flux imbalances often arise due to the varying expression levels of multiple genes within a pathway, leading to the accumulation of metabolic intermediates (Amer and Baidoo 2021).

To identify possible metabolic bottlenecks caused by the different genetic modifications performed on our engineered strains, we conducted a preliminary targeted metabolic study on different compounds related to serotonin and L‐TRP. For this purpose, 20 metabolic compounds from the synthesis of serotonin and derivatives, as well as from the degradation of L‐TRP via the kynurenine pathway, were analysed in BY4743, BS3 and BS4 strains (Table S5). For this analysis, the end‐point samples (72 h; point of maximum production of serotonin) obtained from the serotonin production assay in shake flasks were used, choosing only samples from SD medium without L‐TRP enrichment.

**FIGURE 5:**
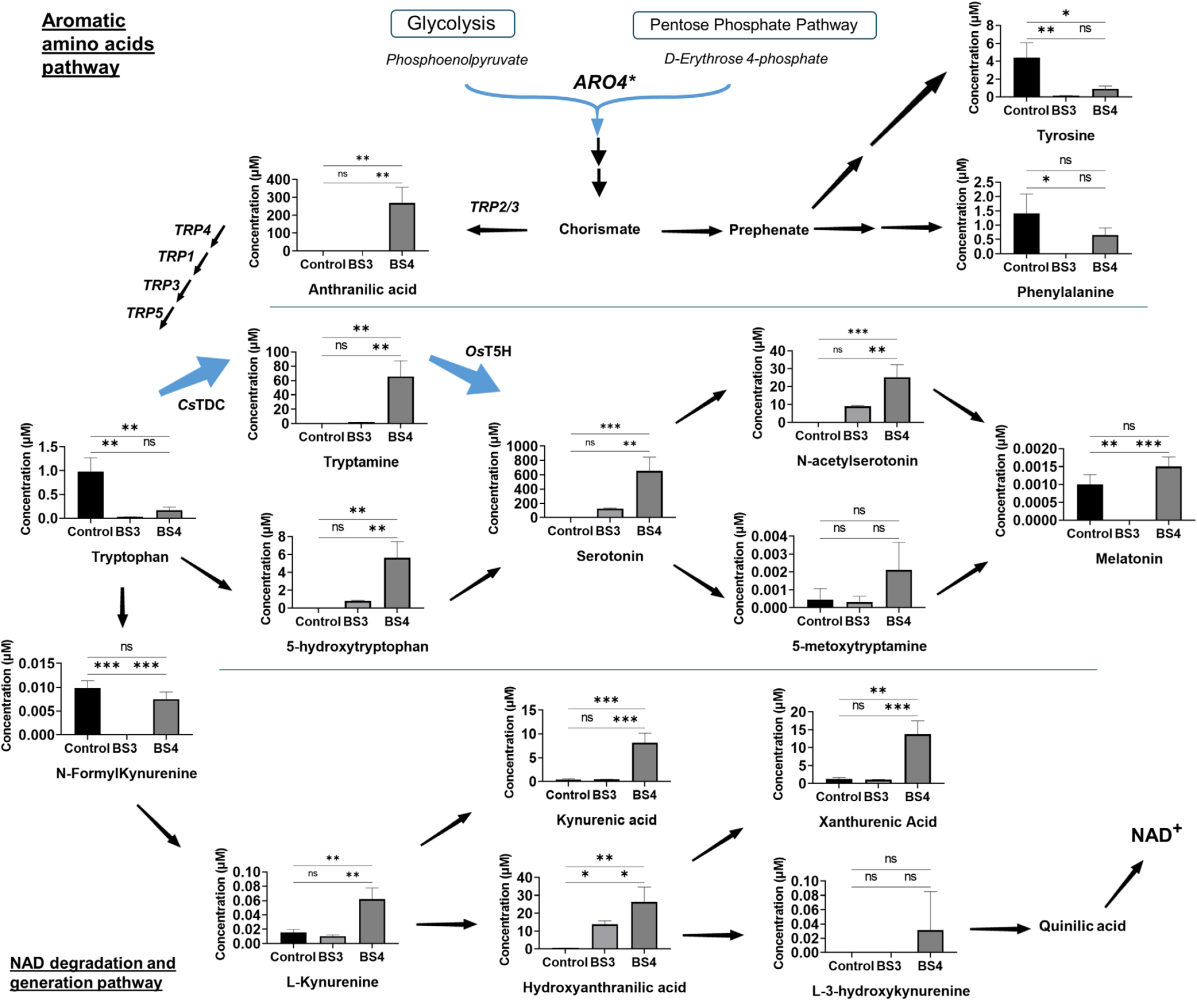
Schematic of tryptophan‐derived metabolic pathways and serotonin production for the study of potential bottlenecks. The strains evaluated are BY4743 (control strain), BS3 strain (harbouring *Cs*TDC and *Os*T5H genes for serotonin production) and BS4 strain (BS3 strain background plus ARO4* gene). Each compound concentration (μM) is showed in a graphic represented by mean and standard deviation. One‐way ANOVA was performance for each compound to show significant differences between strains. The limit of detection (LOD) for each metabolite is given in brackets: Anthranilic acid (10 nM), Tyrosine (2 nM), Phenylalanine (80 nM), Tryptophan (40 nM), Tryptamine (2.5 nM), 5‐hydroxytryptophan (2 nM), Serotonin (10 nM), acetylserotonin (2 nM), 5‐methoxytryptamine (2 nM), Melatonin (5 nM), N‐Formylkynurenine (2 nM), Kynurenine (2 nM), Kynurenic acid (2 nM), Hydroxyanthranilic acid (40 nM), Xanthurenic acid (2 nM), 3‐hydroxykynurenine (2 nM). ns, no significant. *p‐value < 0.05; **p‐value < 0.01; ***p‐value < 0.001. ns = not significant.

Figure 5 shows that serotonin is the major metabolite, with a total of 125 μM (22.00 mg/L) in strain BS3 and up to 657 μM (115.63 mg/L) in strain BS4. The significant increase in serotonin production in strain BS4, which contains *Cs*TDC, *Os*T5H, and ARO4*, compared to strain BS3, which lacks ARO4*, highlights the impact of enhanced precursor supply on serotonin synthesis. Specifically, strain BS4 showed 5.3 times more serotonin than strain BS3. In the case of tryptamine, strain BS3 exhibited a slight increase compared to the control strain. However, strain BS4 accumulated 65 μM of tryptamine, which is 39 times higher than in strain BS3. This substantial accumulation suggests that while the overexpression of ARO4* significantly boosts the precursor supply, the conversion rate of tryptamine to serotonin by *Os*T5H is lower than the conversion of tryptophan to tryptamine by *Cs*TDC. This indicates that *Os*T5H activity may be a limiting factor in our proof‐of‐concept strain. The low expression level and activity of P450s enzymes in yeast have been well documented as major challenges for both fundamental and biotechnological applications (Jiang et al. 2021). To overcome these problems, various strategies have been proposed, such as the expression of P450 and CPR or P450‐CPR fusion proteins, construction of engineered yeast strains to improve the microenvironment for P450, as recently reviewed by Jiang et al. (2021). In our strains containing T5H, we did not overexpress any heterologous CPR but relied on the native activity of the yeast's endogenous CPR encoded by *NCP1*. Compatibility between heterologous P450s and the native CPR in yeast has been previously reported in abscisic acid production (Otto et al. 2019). In this context, *NCP1* was compatible with the *Botrytis cinerea* P450 enzymes; although a 30% reduction in OD_600_ was observed for *NCP1* overexpression compared to the *B. cinerea* CPR strain (Otto et al. 2019). To enhance the conversion of tryptamine to serotonin by *Os*T5H, future strategies may include the combinatorial optimisation of P450–CPR pairing, increasing heme or NADPH pools, or triggering the expansion of the endoplasmic reticulum, which are strategies that have been explored to improve P450 activity in yeast (Arendt et al. 2017; Cheng et al. 2024; Kim et al. 2019). Besides, it would be highly interesting for future research to conduct metabolic flux balance analysis (FBA) to gain a more comprehensive understanding of the metabolic limitations, quantify the identified bottlenecks, and develop other potential targeted strategies to overcome these limitations and enhance the efficiency and productivity of serotonin.

Regarding the downstream products from serotonin, such as N‐acetyl serotonin, 5‐methoxytryptamine and melatonin, we observed the biggest increase of N‐acetyl serotonin in BS4, rising to concentrations of 25 μM. In our previous publication, we have reported that at least *HPA2*, together with *PAA1* in yeast, are involved in the acetylation of tryptamine, serotonin and 5‐methoxytryptamine (Bisquert et al. 2023). This N‐acetyltransferase activity could play a role in the increase in N‐acetyl serotonin observed when elevated serotonin levels are due to overexpression of *Cs*TDC and *Os*T5H. Concerning 5‐methoxytryptamine and melatonin, a slight increase was observed in the BS4 strain compared to BS3, but concentrations reaching around 2 nM, much lower than other metabolites in μM orders. To date, no indole O‐methyltransferase has been described in *S. cerevisiae* , which is the final enzyme in vertebrate melatonin synthesis. Therefore, it remains to be seen whether the enzymes involved in melatonin synthesis in yeasts are exclusive to this process or, as already mentioned by Ganguly et al. (2001), the synthesis of this molecule may reflect the opportunistic action of otherwise unrelated metabolic enzymes with broad functions.

It should also be noted that the increased metabolic flux in the shikimic acid pathway does not affect other aromatic amino acids, such as L‐tyrosine or L‐phenylalanine, even though these showed a slight decrease (Figure 5). It may be explained by the fact that the presence of the *TDC* and *T5H* genes forces the metabolic flux towards serotonin and allows the other amino acids to keep their concentrations in balance.

In the case of metabolites from L‐TRP catabolism, there is some accumulation of acids, with kynurenic acid increasing to 8.12 μM, xanthurenic acid to 13.78 μM and 3‐hydroxyanthranilate acid to 26.42 μM. One of the most striking effects of multi‐copy integration of ARO4* is the accumulation of anthranilic acid, reaching 268.83 μM. This could come from increased metabolic flux to the shikimic acid pathway, being a product of the enzyme anthranilate synthase (*TRP2*), but may also be derived from the degradation of L‐kynurenine to produce alanine. Anthranilate is a precursor molecule for L‐TRP, so increasing the expression of genes downstream of this molecule would unblock this bottleneck and increase the flow towards L‐TRP, and consequently towards a higher production of serotonin. Some of the genes that would be interesting to test for overexpression are *TRP4*, *TRP1*, *TRP3* and *TRP5*.

## 4 Conclusion and Future Outlook

Society's increasing demand for healthier lifestyles and longer life expectancy is challenging the food and pharmaceutical industries to find health‐promoting, natural and effective sources for drugs and food ingredients. Some of the molecules derived from yeast aromatic amino acid metabolism have recently attracted much attention for their health benefits. Serotonin is an L‐TRP‐derived molecule that plays an important role in mammals as a neurotransmitter that plays an essential regulatory role in many cognitive and behavioural functions, as well as in communication and regulation of the gut microbiota. Serotonin can be obtained via chemical synthesis or extraction from animals or plants; however, present challenges, such as environmental issues and the use of unsustainable materials, have limited the yields of serotonin to far below the theoretical maximum output (Shen et al. 2022). Therefore, a simple biotechnological method for the production of these compounds is desirable. Several previous studies have developed a metabolically engineered *E. coli* platform for high‐level serotonin production from L‐TRP (Li et al. 2024; Shen et al. 2022; Wang et al. 2022), mainly by heterologous overexpression of the vertebrate enzymes, which have the disadvantage of using cofactors that are not available in the bacteria. In our study, we followed a very novel strategy by using a bacterial gene that has not yet been used for serotonin synthesis and a plant gene that requires NADPH to carry out the enzymatic transformation. Our results demonstrated a high conversion rate of L‐TRP to tryptamine by a *C. sporogenes* decarboxylase by integrating and overexpressing this bacterial gene into the *S. cerevisiae* genome. We also demonstrated an efficient hydroxylation rate of tryptamine into serotonin by the T5H of *O. sativa* and, what we consider very important in terms of reducing the metabolic burden of the constructed strain, this reaction was uncoupled from the endogenous vital cofactor tetrahydrobiopterin (BH4) for efficient serotonin production. As expected, overexpression of the feedback‐resistant version of ARO4* significantly increased the flux of the shikimate pathway and the titres of serotonin using the carbon skeleton directly derived from glucose, from the order of 0.352 mg/L of the control strain to 139 mg/L, which represented around a 400‐fold increase. The optimisation of the growth medium and fermentation conditions resulted in the highest concentration of serotonin reported so far for a yeast strain (250 mg/L), although this concentration can be increased by some specific improvements in the current strain, as evidenced by metabolomic analysis.

It is well known that *S. cerevisiae* is one of the preferred chassis for the biotechnology industry as a cell factory for the production of recombinant molecules, thanks to a number of advantages such as robust growth on simple media, feasibility of genetic manipulation and being “generally regarded as safe” (GRAS) (Guo et al. 2019; Nielsen et al. 2013). We have constructed a *S. cerevisiae* strain that represents the first steps towards a feasible industrial production of serotonin through a sustainable and environmentally friendly approach, paving the way for the development of similar biotechnological strategies in the future. Future studies using metabolic flux balance analysis (FBA), enzyme improvement through protein engineering, and other approaches to enhance the functional expression of P450s in yeast could significantly improve the biotechnological production of serotonin.

## Author Contributions

**Andrés Planells‐Cárcel:** conceptualization, methodology, formal analysis, visualization, validation, investigation, writing – original draft. **Elena Valera‐García:** validation, investigation, methodology. **Guillermo Quintas:** investigation, validation, visualization. **José Luis Martínez:** methodology, supervision. **Sara Muñiz‐Calvo:** conceptualization, methodology, investigation, writing – review and editing, supervision, validation. **José Manuel Guillamón:** funding acquisition, project administration, supervision, writing – review and editing, resources.

## Conflicts of Interest

The authors declare no conflicts of interest.

## Supporting information


Figure S1.

Figure S2.

Figure S3.

Figure S4.

Table S1.
Table S2.
Table S3.
Table S4.
Table S5.

## Data Availability

The data that supports the findings of this study are available in the Supporting Information of this article.
