# Supplementary figures and images for: Metabolic Engineering of a Serotonin Overproducing Saccharomyces cerevisiae Strain
Source: Microb Biotechnol. 2025 Apr 5;18(4):e70140. doi: 10.1111/1751-7915.70140 (PMC11971721; doi:10.1111/1751-7915.70140)

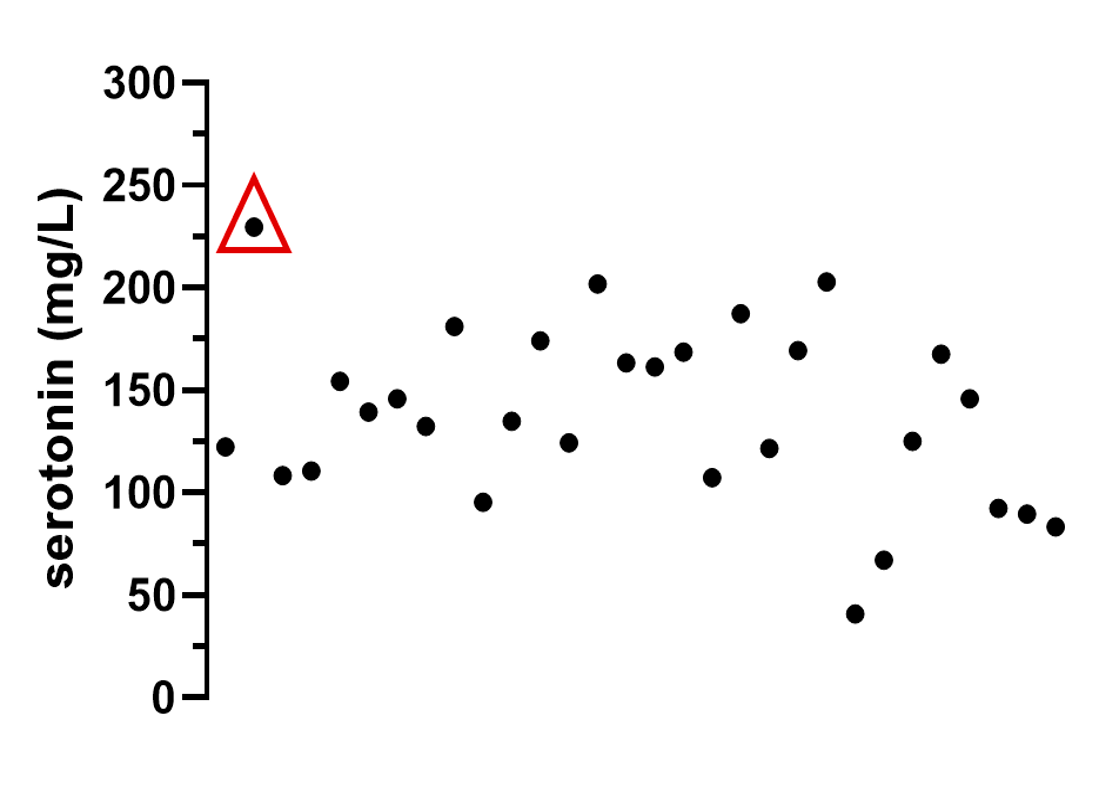

Supplement: Supplementary file 1 — Figure S1. [file MBT2-18-e70140-s002.png]

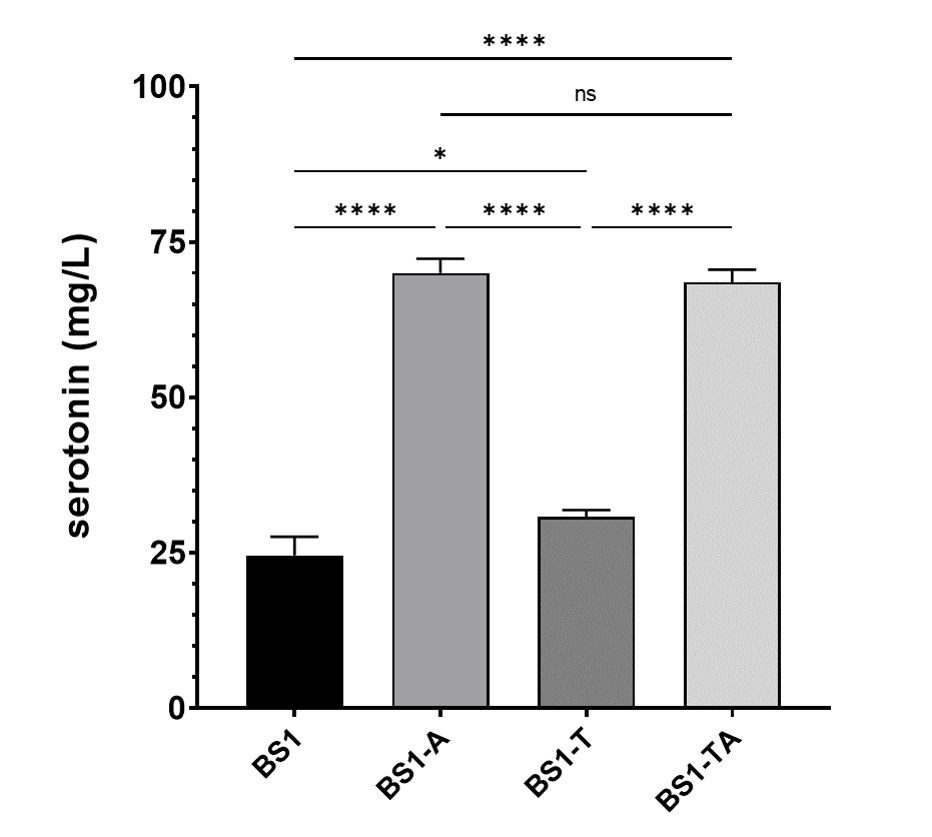

Supplement: Supplementary file 2 — Figure S2. [file MBT2-18-e70140-s001.png]

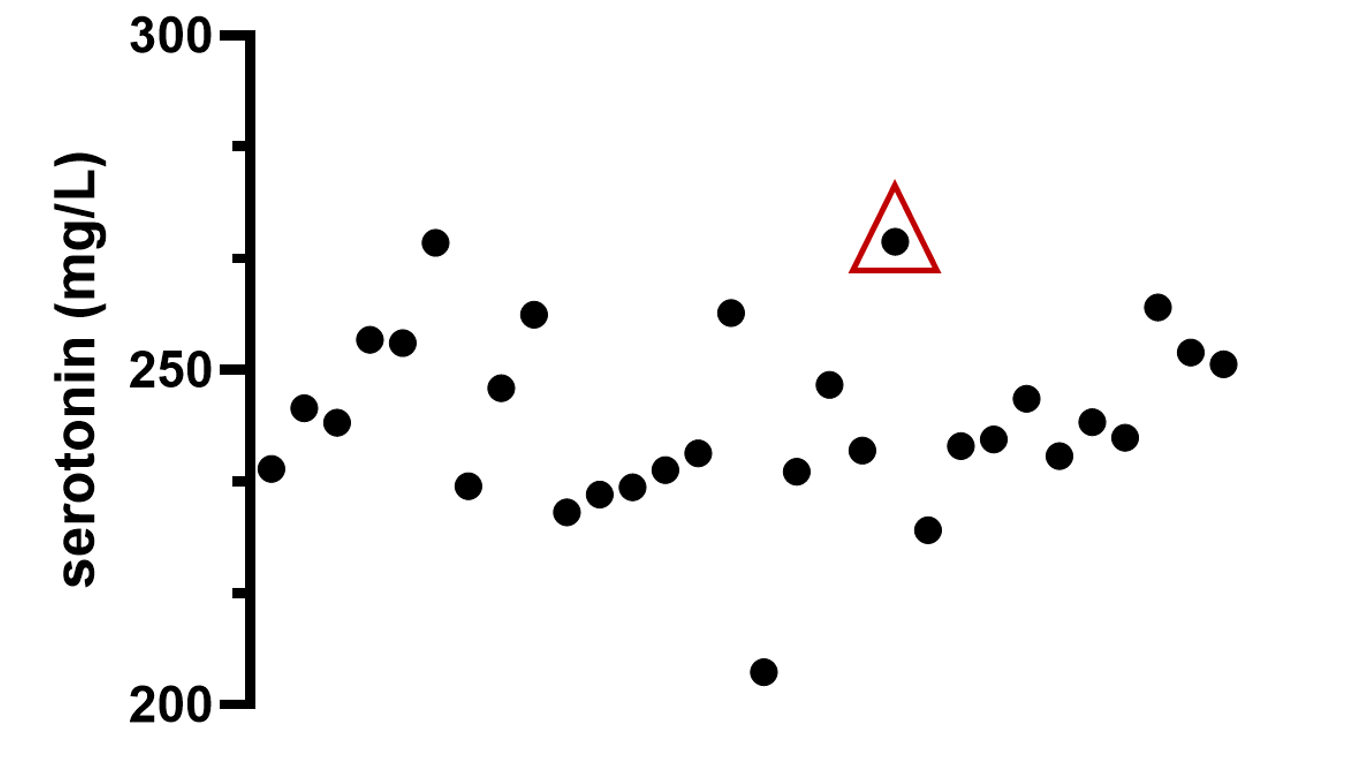

Supplement: Supplementary file 3 — Figure S3. [file MBT2-18-e70140-s004.png]

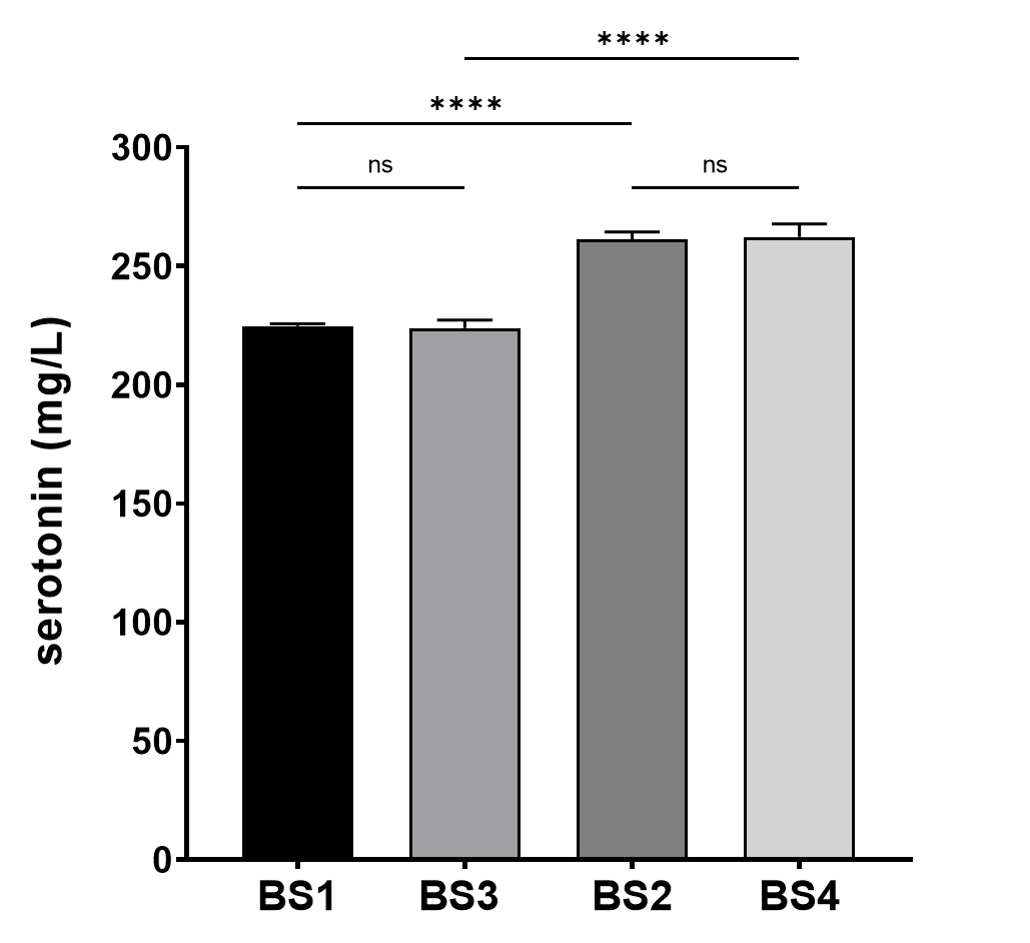

Supplement: Supplementary file 4 — Figure S4. [file MBT2-18-e70140-s005.png]
