# Supplementary material for: Metabolic Engineering of a Serotonin Overproducing Saccharomyces cerevisiae Strain
Source: Microb Biotechnol. 2025 Apr 5;18(4):e70140. doi: 10.1111/1751-7915.70140 (PMC11971721; doi:10.1111/1751-7915.70140)
Supplement: Supplementary file 5 — Table S1. Table S2. Table S3. Table S4. Table S5. [file MBT2-18-e70140-s003.docx]

**SUPPLEMENTARY TABLES**

**Supplementary Table 1**. List of the strains used in this study.

| **Strain name** | **Parental strain** | **Genomic modifications** | **Plasmid** | **Source** |
| --- | --- | --- | --- | --- |
| BY4743 | - | MATa/α *his3∆1/his3∆1* *leu2∆0*/*leu2∆0* *met15∆0*/*MET15* *LYS2*/*lys2∆0* *ura3∆0*/*ura3∆0* | - | Euroscarf |
| BS1 | BY4743 | TEF1p::*Cs*TDC; PGK1p::*Os*T5H, *LEU2* | - | This study |
| BS1-T | BS1 | - | p426GPD TRP2* | This study |
| BS1-A | BS1 | - | p423GPD ARO4* | This study |
| BS1-TA | BS1 | - | p426GPD TRP2*  p423GPD ARO4* | This study |
| BS2 | BS1 | TDH3p::*ARO4^K229L^ , HIS3* | - | This study |
| BS3 | BS1 | *URA3, HIS3* | - | This study |
| BS4 | BS2 | *URA3* | - | This study |

**Supplementary Table 2**. List of the plasmids used in this study.

| **Plasmid name** | **Description** | **Source** |
| --- | --- | --- |
| p426GPD | Multicopy vector (*URA3* marker, GPD promoter) | *(Mumberg et al., 1995)* |
| p426GPD-CsTDC | p426GPD with the *TDC* gene from *C. sporogenes*, Amp^R^ | This work |
| p426GPD-OsT5H | p426GPD with the *T5H* gene from *O. sativa,* Amp^R^ | This work |
| pCfB2803 | Integrative vector for multiple integrations at sites sharing homology with Ty4Cons (Kl.LEU2-deg marker) | (Maury et al., 2016) |
| pCfB2803 TDC + T5H | pCfB2803 with TEF1p::*Cs*TDC; PGK1p::*Os*T5H | This work |
| pCfB2797 | Integrative vector for multiple integrations at sites sharing homology with Ty2Cons (Kl.URA3-deg marker) | (Maury et al., 2016) |
| pCfB2797 HIS3 | Integrative vector for multiple integrations at sites sharing homology with Ty2Cons (Kl.HIS3-deg marker) | This work |
| p423GPD ARO4* | p423GPD with *ARO4^K229L^* gene from *S. cerevisiae* | (Bisquert et al. 2021) |
| p426GPD TRP2 | p426GPD with *TRP2* gene from *S. cerevisiae* | This work |
| p426GPD TRP2* | p426GPD with *TRP2^S65R,S76L^* gene from *S. cerevisiae* | This work |
| pCfB2797 HIS3 ARO4* | pCfB2797 HIS3 with TDH3p::*ARO4^K229L^* | This work |
| pCfB2988 | Integrative vector for multiple integrations at sites sharing homology with Ty1Cons (Kl.URA3-deg marker) | (Maury et al., 2016) |
| pCfB2628 | Bi-directional promoter TEF1p-PGK1p | (Germann et al., 2016) |

**Supplementary Table 3**. List of the oligonucleotides used in this study.

| **Primer name** | **Sequence 5’→3’** |
| --- | --- |
| CsTDC BamHI F | CCGCGGATCCATGAAATTTTGGAGGA |
| CsTDC XhoI R | CGCGCTCGAGTTACATTGCTTTTAAGTG |
| OsT5H BamHI F | CCGCGGATCCATGGAATTGACAATG |
| OsT5H XhoI R | CGCGCTCGAGTTAGACTTCCGATAA |
| ARO4 BamHI F | GTCGTGGGATCCAAATGAGTGAATCTCCAATGTTCGCTGCCAAC |
| ARO4 K229L R | GCAGCAACACCATGCAAAGTAACACCCATG |
| ARO4 R XhoI R | CCGCTCGAGCTATTTCTTGTTAACTTCTCTTCTTTGTCTGACAGC |
| ARO4 K229L F | CATGGGTGTTACTTTGCATGGTGTTGCTGC |
| TRP2 BamHI F | TGGGATCCTTATGACCGCTTCCATCAAAAT |
| TRP2 XhoI R | CCGCTCGAGCTTTTTAAGCTGATCCTACGA |
| TRP2 S65R F | ATCATTTCTGTTGGAAcGTGCTAAGACAAATAA |
| TRP2 S65R R | TTATTTGTCTTAgCACGTTCCAACAGAAATGAT |
| TRP2 S76L F | GAATTAGATCGTTATTtATTCATAGGTATCTCG |
| TRP2 S76L R | CGAGATACCTATGAATaAATAACGATCTAATTC |
| PV2F (GPDp) | CGTGCGAUAGTTTATCATTATCAATACTCGCC |
| GV2R (ARO4) | CACGCGAUCTATTTCTTGTTAACTTCTCTTCTTTGTCT |
| CsTDC-GV1R | CGTGCGAUTTACATTGCTTTTAAGTGTTCCT |
| CsTDC-GP1F | AGTGCAGGUAAAACAATGAAATTTTGGAGGAAATATACAC |
| OsT5H-GV2R | CACGCGAUTTAGACTTCCGATAATTCTTCCCC |
| OsT5H-GP2F | ATCTGTCAUAAAACAATGGAATTGACAATGGCTTCTAC |
| PG1R(TEF1p) | ACCTGCACUTTTGTAATTAAAACTTAGATTAGATTGCTAT |
| PG2R (PGK1p) | ATGACAGAUTTGTTTTATATTTGTTGTAAAAAGTAGATAATT |
| ADH1_test_fw | GAAATTCGCTTATTTAGAAGTGTC |
| CYC1_test_rv | CTCCTTCCTTTTCGGTTAGAG |
| GPD_test_fw | CGGTAGGTATTGATTGTAATTCTG |

**Supplementary Table 4**. List of synthesised codon-optimised genes with used in this study.

| **Gene name** | **Sequence 5’→3’** |
| --- | --- |
| CsTDC | ATGAAATTTTGGAGGAAATATACACAACAAGAGATGGATGAGAAAATTACAGAAAGCTTAGAGAAAACCCTTAACTACGATAACACCAAAACAATAGGTATCCCAGGAACTAAATTGGATGATACCGTCTTTTATGATGACCATAGTTTTGTGAAGCATTCACCCTATTTGAGAACGTTCATACAGAATCCAAATCATATTGGCTGTCATACCTACGATAAAGCCGACATTCTGTTTGGAGGTACTTTCGACATTGAGAGAGAGTTGATTCAACTTCTAGCGATAGATGTCCTAAATGGCAACGATGAAGAATTTGACGGGTATGTTACTCAAGGTGGTACAGAAGCCAATATTCAAGCAATGTGGGTTTATAGAAACTACTTCAAGAAGGAGAGAAAAGCTAAACACGAAGAAATTGCCATTATTACTTCTGCGGATACACACTATTCTGCATACAAAGGTAGTGATTTACTAAACATAGACATCATCAAAGTACCAGTAGACTTTTACTCTAGAAAGATCCAGGAAAATACATTGGATTCAATCGTTAAGGAAGCAAAGGAAATTGGTAAGAAATATTTTATCGTCATTAGCAATATGGGCACTACGATGTTTGGGTCAGTAGATGATCCAGATCTTTATGCCAATATCTTTGACAAATACAACTTGGAGTATAAGATTCATGTTGATGGTGCTTTTGGCGGATTTATATATCCGATTGACAACAAAGAATGCAAAACTGATTTCTCCAATAAGAATGTTTCCTCCATAACTCTGGATGGTCACAAGATGTTACAAGCTCCTTACGGAACTGGGATATTCGTGAGTCGTAAGAATTTGATTCATAATACCTTAACGAAAGAAGCTACCTATATTGAGAACTTGGACGTCACTTTATCTGGTTCAAGATCTGGATCAAATGCTGTTGCAATTTGGATGGTCTTAGCTTCGTATGGTCCTTATGGTTGGATGGAGAAGATTAACAAATTGAGAAACAGAACGAAATGGTTGTGTAAGCAACTGAATGATATGCGTATAAAATACTATAAGGAAGATTCTATGAATATCGTAACAATCGAAGAACAGTACGTGAACAAGGAAATCGCTGAAAAGTACTTCTTAGTTCCTGAAGTTCATAACCCCACAAATAATTGGTATAAAATAGTGGTTATGGAACATGTAGAACTAGATATACTGAATTCGCTTGTGTACGACTTGAGGAAATTCAATAAGGAACACTTAAAAGCAATGTAA |
| OsT5H | ATGGAATTGACAATGGCTTCTACAATGTCACTGGCTCTGTTAGTTTTATCCGCAGCATATGTCTTAGTTGCATTGAGAAGATCTAGAAGTAGCTCTTCGAAGCCGCGTAGATTGCCACCATCTCCACCAGGTTGGCCAGTTATTGGGCATTTGCATTTGATGTCAGGCATGCCTCATCATGCTTTAGCTGAACTAGCGAGGACAATGAGAGCTCCACTATTCAGAATGAGGTTGGGTTCTGTTCCTGCAGTTGTGATTAGTAAACCCGATTTAGCGAGGGCAGCATTAACGACAAATGACGCAGCCTTGGCTTCACGTCCCCATCTTCTTAGTGGTCAGTTCCTATCCTTCGGATGCTCGGATGTAACATTCGCTCCAGCTGGTCCATACCACAGAATGGCTAGAAGAGTTGTGGTGTCTGAGCTATTAAGCGCCAGACGTGTTGCCACTTATGGAGCGGTTAGAGTAAAGGAGCTAAGGAGATTATTGGCTCATTTGACTAAGAATACTAGTCCCGCAAAACCCGTAGACTTATCCGAATGCTTTCTGAACTTGGCAAATGATGTCCTATGTAGAGTGGCATTTGGGAGAAGGTTTCCTCATGGTGAAGGAGATAAATTGGGAGCAGTTTTAGCTGAAGCCCAAGATTTGTTTGCTGGCTTTACGATCGGCGATTTCTTTCCGGAATTAGAGCCTGTGGCCTCAACTGTTACTGGCTTGAGGAGAAGGTTGAAGAAGTGCCTAGCTGACTTAAGAGAGGCTTGTGACGTTATAGTCGATGAACACATTAGCGGTAATAGGCAAAGGATACCAGGCGATAGAGATGAGGACTTCGTAGATGTGCTGTTGCGTGTGCAGAAATCCCCTGATTTAGAAGTACCTCTGACTGATGATAACCTTAAGGCCTTGGTACTAGACATGTTCGTAGCTGGCACAGATACAACCTTTGCTACCTTGGAATGGGTTATGACCGAACTTGTCAGACATCCGAGAATTTTGAAGAAAGCCCAAGAAGAAGTGAGAAGAGTTGTAGGTGATAGCGGTAGAGTTGAAGAATCACACTTAGGAGAGCTGCACTACATGAGAGCTATCATCAAAGAAACCTTCAGACTGCATCCTGCAGTCCCTTTACTTGTACCTAGAGAATCAGTCGCTCCTTGTACTTTGGGTGGTTATGACATTCCAGCAAGAACTCGTGTTTTCATTAACACGTTCGCTATGGGTAGAGATCCAGAGATATGGGATAATCCATTGGAATATTCACCGGAGAGGTTTGAGTCTGCAGGAGGAGGTGGGGAAATAGACCTTAAAGATCCAGACTACAAATTGTTGCCTTTTGGTGGTGGTAGACGTGGCTGTCCAGGTTACACATTTGCGTTAGCGACTGTCCAAGTTAGTCTTGCCTCCCTTCTTTATCACTTTGAATGGGCCTTACCAGCCGGAGTTAGAGCCGAAGATGTCAACTTGGACGAAACCTTTGGTCTAGCAACCCGTAAGAAAGAGCCCCTATTTGTTGCGGTGAGAAAATCTGATGCCTATGAATTTAAAGGGGAAGAATTATCGGAAGTCTAA |

**Supplementary Table 5.** Values of each metabolite (µM) measured by UPLC-MSMS on the metabolic indole analysis for each of the strains: Control (BY4743), BS3 and BS4.

|  | ***Anthranilic acid (µM)*** | ***3-Hydroxyanthranilic acid (µM)*** | ***Tryptamine (µM)*** | ***L-Phenylalanine (µM)*** | ***Serotonin (µM)*** | ***Kynurenic acid (µM)*** | ***5-Methoxytryptamine (µM)*** | ***L-Tryptophan (µM)*** |
| --- | --- | --- | --- | --- | --- | --- | --- | --- |
| *Control* | 1,8145 | 0,4944 | 0,3052 | 1,0785 | 3,3524 | 0,3902 | 0,0012 | 0,8760 |
| *Control* | 1,0216 | 0,5913 | 0,1445 | 2,1878 | 1,8030 | 0,5870 | 0,0002 | 1,3074 |
| *Control* | 0,5961 | 0,4259 | 0,0839 | 0,9778 | 0,9948 | 0,2806 | 0,0000 | 0,7771 |
| *BS3* | 0,3658 | 13,8147 | 1,5458 | 0,0000 | 123,7478 | 0,5053 | 0,0003 | 0,0329 |
| *BS3* | 0,1886 | 11,8149 | 1,8009 | 0,0000 | 118,9097 | 0,4650 | 0,0007 | 0,0275 |
| *BS3* | 0,1330 | 15,7846 | 1,7189 | 0,0000 | 134,3995 | 0,4953 | 0,0000 | 0,0225 |
| *BS4* | 350,4948 | 29,9140 | 83,7141 | 0,7461 | 793,5168 | 9,0569 | 0,0034 | 0,2370 |
| *BS4* | 280,0777 | 32,2804 | 71,6480 | 0,8430 | 738,7747 | 9,5194 | 0,0026 | 0,1638 |
| *BS4* | 175,9162 | 17,0681 | 41,0505 | 0,3672 | 438,8185 | 5,7985 | 0,0004 | 0,1104 |

|  | ***Xanthurenic acid*** | ***L-Kynurenine*** | ***N-acetylserotonin*** | ***5-Hydroxy-L-tryptophan*** | ***L-3-Hydroxykynurenine*** | ***Melatonin*** | ***N'-Formylkynurenine*** | ***L-Tyrosine*** |
| --- | --- | --- | --- | --- | --- | --- | --- | --- |
| *Control* | 1,2480 | 0,0147 | 0,1532 | 0,0207 | 0,0000 | 0,0011 | 0,0094 | 3,7599 |
| *Control* | 1,7285 | 0,0200 | 0,0762 | 0,0105 | 0,0000 | 0,0007 | 0,0116 | 6,2897 |
| *Control* | 0,9707 | 0,0111 | 0,0370 | 0,0082 | 0,0000 | 0,0013 | 0,0086 | 3,1967 |
| *BS3* | 1,0435 | 0,0086 | 8,8673 | 0,8000 | 0,0000 | 0,0000 | 0,0000 | 0,1377 |
| *BS3* | 0,8831 | 0,0108 | 8,5428 | 0,7663 | 0,0000 | 0,0000 | 0,0000 | 0,1551 |
| *BS3* | 1,1148 | 0,0113 | 9,4496 | 0,8637 | 0,0000 | 0,0000 | 0,0000 | 0,1414 |
| *BS4* | 15,1778 | 0,0695 | 30,9107 | 6,4471 | 0,0937 | 0,0014 | 0,0059 | 1,1847 |
| *BS4* | 16,6150 | 0,0724 | 27,4662 | 6,9006 | 0,0000 | 0,0018 | 0,0079 | 0,9611 |
| *BS4* | 9,5454 | 0,0441 | 17,3122 | 3,5208 | 0,0000 | 0,0013 | 0,0088 | 0,5717 |
